# Prize Offered by the American Medical Association

**Published:** 1850-10

**Authors:** 


*Prize offered by the American Medical Association.*—The following resolution, appended to the Report of the *Committee on Medical Literature,* was adopted by the Association at the meeting in Cincinnati in May last.

*Resolved,* That the sum of ONE HUNDRED DOLLARS, raised by voluntary contribution, be offered by this Association for the best *experimental* essay on a subject connected either with PHYSIOLOGY, or MEDICAL CHEMISTRY, and that a committee of seven be appointed to carry out the objects of this resolution: Said committee to receive the competing memoirs until the first day of March, 1851; the author's names to be concealed from the committee; and the name of the successful competitor alone to be announced after the publication of the decision.

Dr. FRANCIS G. SMITH, Philadelphia, Chairman.

Dr. ALFRED STILLE, Philada. Dr. JAMES MOULTRIE, Charleston, S. C.
" FRANKLIN BACHE, " " ROBERT BRIDGES, Philada.
" L. P. YANDELL, Louisville, Ky. " WASHINGTON L. ATLEE, Philada.

In accordance with the above resolution, the Chairman gives notice that the sum of *one hundred dollars* is secured, and will be paid over to the successful competitor, or, if preferred, a gold medal of equal value bearing a suitable inscription.

The competing memoirs must be transmitted to the chairman, free of expense, and should be designated by some appropriate motto; the author's name accompanying it in a sealed packet, designated in like manner. The successful essay will become the property of the Association, and in case no paper of sufficient merit is offered, the time will be extended for another year.

After the decision of the committee, the sealed packet containing the author's name will be opened in the presence of the Association.

Medical Journals throughout the country are requested to give publicity to the above notice, and to aid in furthering the wishes of the Association in this respect.

Francis G. Smith, M. D, Philada., *Chairman.*

